# Radial aplasia with oligodactyly

**DOI:** 10.4103/0971-6866.42326

**Published:** 2008

**Authors:** Panigrahi Inusha, Kulkarni Ketan Prasad

**Affiliations:** Genetic and Metabolic Unit, Department of Pediatrics, Advanced Pediatric Center, PGIMER, Chandigarh, India

**Figure 1:**
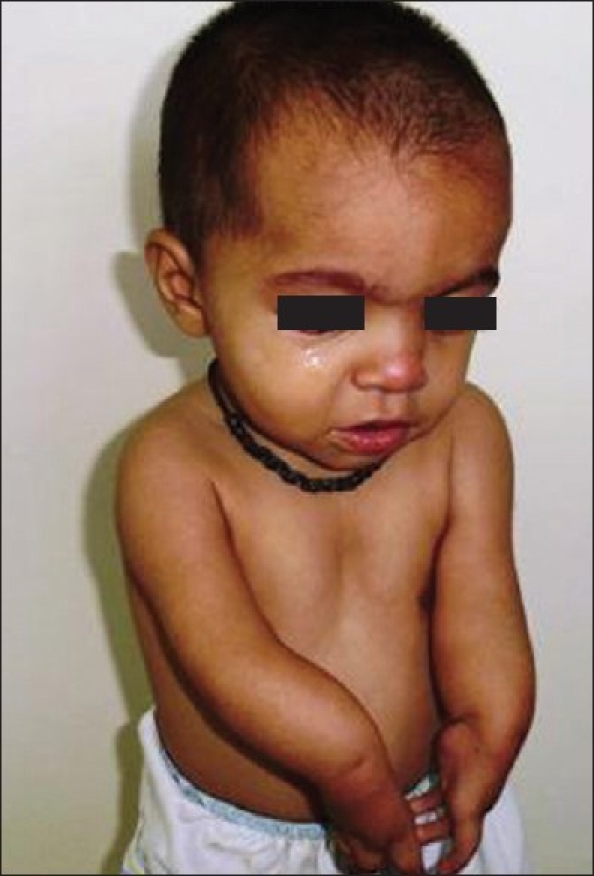
Face and hands showing no significant dysmorphism, mesomelic shortening of upper limbs, and oligodactyly

Sir,

A 15-month-old female child presented with bilateral upper limb deformity. She was born of a non-consanguineous marriage between a 23-year-old mother and 25-year-old father and was delivered by a normal vaginal delivery at 39 weeks of gestation. There was no history of drug intake in early gestation or of antenatal radiation exposure. On examination, there was mesomelic shortening of the forearms, skin dimpling, and oligodactyly, with absence of movement at the elbows [Figure 1 and 2a]. There were no other dysmorphic features, and the child was developmentally and neurologically normal. The platelet count was 274.0 × 10^9^/L. The skiagram of upper limbs revealed humeroulnar fusion, ulnar campomelia, absent radius, and oligodactyly [Figure 2b].

**Figure 2a:**
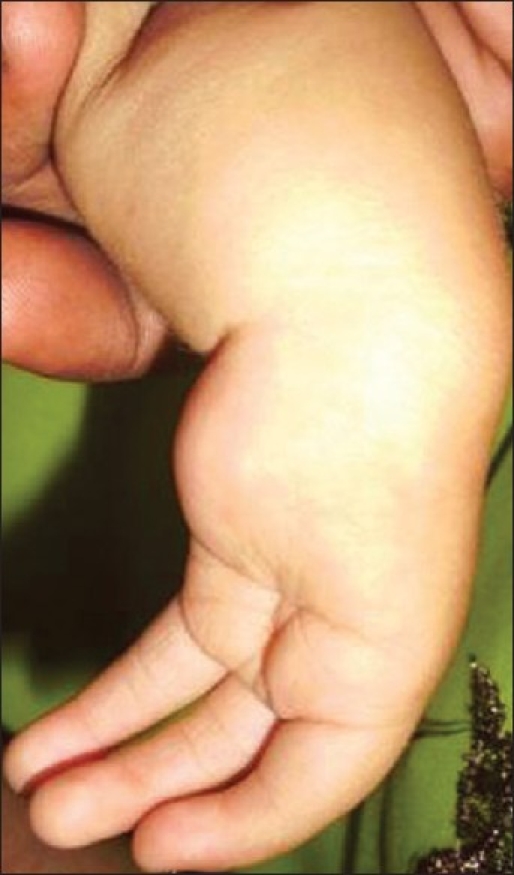
Left upper limb showing short forearm, small hand, absence of thumb, and presence of only three fingers

**Figure 2b:**
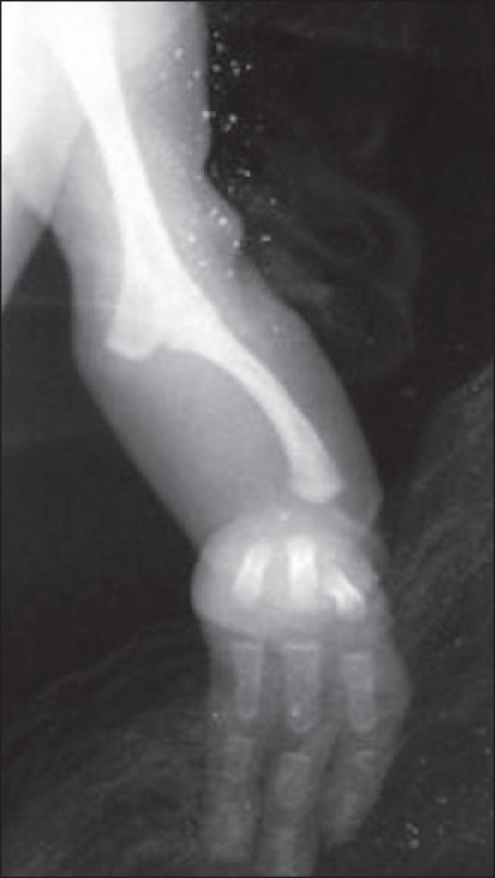
X-ray of the upper limb revealing absence of radius, bowed ulna, and humeroulnar synostosis

Radial aplasia with oligodactyly has been described in the Al-Awadi / Rothschild syndrome / Schinzel phocomelia syndrome which has a wide clinical spectrum.[1] However, humeroradial and humeroulnar synostosis has been described only in the Schinzel phocomelia spectrum of disorders.[2] Hence, our patient is either a variant of this syndrome or an entirely new entity. Mutations in WNT7A, HOXD13, and GLI3 genes have been described in the genesis of such limb malformations.[3] In view of the clinical variability in the presentation of such complex limb reduction defects, their varied prognosis, and the need for multidisciplinary management, it is essential to provide appropriate genetic counseling. Antenatal diagnosis can be offered to the families in selected situations.
